# Mixed genotypes of *Orientia tsutsugamushi* in conserved genes and a single immune-dominant *tsa56* genotype discovered from a patient with scrub typhus in Hainan Island, China: a case report

**DOI:** 10.1186/s12879-022-07682-y

**Published:** 2022-08-19

**Authors:** Chuanning Tang, Liyuan Zhang, Yi Huang, Wenhui Mai, Liying Xue, Gaoyu Wang, Shu Wen, Ruoyan Peng, Kunliang Wu, Xiuying Tian, Hua Pei, Jiang Du, Kwok-Yung Yuen, Jasper Fuk-Woo Chan, Yongguo Du, Feifei Yin

**Affiliations:** 1 Key Laboratory of Tropical Translational Medicine of Ministry of Education, Hainan Medical University, Haikou, 571199 China; 2 The University of Hong Kong Joint Laboratory of Tropical Infectious Diseases, Hainan Medical University, Haikou, Hainan China; 3 Department of Pathogen Biology, Hainan Medical University, Haikou, Hainan China; 4 Academician Workstation of the Hainan Province, Hainan Medical University, Haikou, 571199 Hainan China; 5 Department of Infectious Disease, The Second Affiliated Hospital of Hainan Medical University, Haikou, China; 6 Haikou Maternal and Child Health Hospital, Haikou, 571199 Hainan China; 7 Clinical Laboratory, People’s Hospital of Qiongzhong Li Miao Autonomous County, Qiongzhong, Hainan China; 8 State Key Laboratory of Emerging Infectious Diseases, Carol Yu Center for Infection, Department of Microbiology, Li Ka Shing, Faculty of Medicine, The University of Hong Kong, Pokfulam, Hong Kong Special Administrative Region China; 9 Department of Clinical Microbiology and Infection Control, the University of Hong Kong-Shenzhen Hospital, Shenzhen, Guangdong China

**Keywords:** Scrub typhus, *Orientia tsutsugamushi*, Mixed-infections, *tsa56*, Multi-locus sequence typing

## Abstract

**Background:**

*Orientia tsutsugamushi* (*O. tsutsugamushi*), an obligate intracellular bacterium, is transmitted to humans through infected larval trombiculid mite bites, causing scrub typhus. Mixed genotypes of *O. tsutsugamushi* in canonical conserved genes were reported in 8–25% of blood samples from patients. Yet, there are few clinical descriptions of these mixed *O. tsutsugamushi*-infected patients.

**Case presentation:**

We report a patient with scrub typhus complicated with pulmonary involvement and hepatic dysfunction, who carried mixed genotypes of the conserved genes but had a single immune-dominant 56-kDa type-specific antigen (*tsa56)* genotype. The patient was successfully recovered by doxycycline treatment.

**Conclusions:**

In this reported case, both patient’s eschar and blood samples have repeatedly shown the same results, i.e., no variants were discovered in *tsa56* gene that bears multiple hypervariable regions. Whereas the selected highly conserved genes were identified with up to 32 variants in a 2700 base-pair concatenated sequence. The prevalence, disease severity and mechanism of these single-tsa56-genotype mixed infections remain to be investigated on a large scale with more cases.

**Supplementary Information:**

The online version contains supplementary material available at 10.1186/s12879-022-07682-y.

## Background

*Orientia tsutsugamushi* (*O. tsutsugamushi*) is an obligate intracellular bacterium that causes scrub typhus in humans via bites by infected larval trombiculid mites. Scrub typhus is a leading cause of severe febrile illness in the Asia–Pacific tsutsugamushi triangle and, more recently, in the Middle East and South America [1, 2]. Interestingly, mixed genotypes of *O. tsutsugamushi* in canonical genes were discovered in 8–25% of blood samples from patients [3, 4], whereas few cases of co-infections with mixed *tsa56* genotypes have been reported in patients [3–7]. These findings have suggested a rather distinct inconsistency in the discovery rates of mixed genotypes *O. tsutsugamushi* infections between the conserved genes and the 56-kDa type-specific antigen (*tsa56*) gene of *O. tsutsugamushi*. Consequently, this inconsistency limits our understanding of *O. tsutsugamushi* co-infections. In addition, clinical characteristics of *O. tsutsugamushi* mixed-infections of conserved genes have not been described in details. Here, we report a patient with scrub typhus who was infected by an unreported mixed-infection, i.e., mixed genotypes being discovered in the highly conserved genes’ region, including several novel genotypes, but only being shown one *tsa56* genotype of the *O. tsutsugamushi* strains. Both patient’s eschar and blood samples have shown the same results, suggesting consistency of evaluations on the discovery and the *O. tsutsugamushi* strains originated from the carrying host.

## Case presentation

**Fig. 1:**
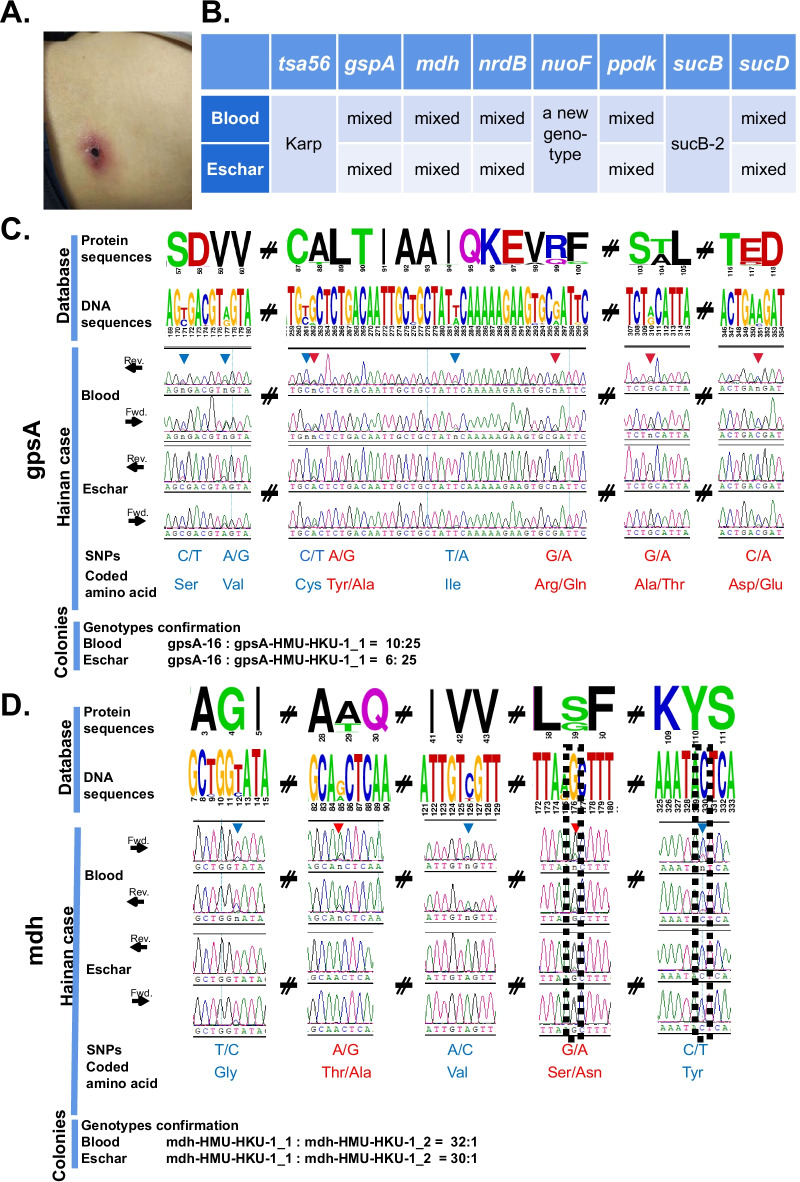
Mixed genotypes of *O. tsutsugamushi* identified from both eschar and blood samples of the patient. **A** A crater-shaped eschar (5 × 5 mm) was discovered on the left waist of the patient. **B** Five of the seven conserved genes showing mixed genotypes of *O. tsutsugamushi* in the patient’s eschar and blood samples. **C**, **D** The results of direct sequencing of PCR amplicon and colony verification of the *gpsA* and *mdh* genes. The logo presents the nested PCR fragments of *gpsA* and *mdh* genes collected in the pubMLST database. Mixed sites are indicated by filled arrows. These mixed sites are either synonymous (light blue letters) or missense (red letters). Two novel SNP sites in the *mdh* gene are also indicated by the unfilled line. These sites are all verified by sequencing of 30–40 clones for each gene and each sample

A sixty-year-old male farmer, residing on the northern coast of Hainan Island, was admitted to the Second Affiliated Hospital of Hainan Medical University on December 8, 2018. He presented with a high fever (38.4–40.0 °C), chills, headache, myalgia, and cough for six days. A typical 5 × 5 mm eschar was observed on the lateral side of the left waist (Fig. 1A). Laboratory examination showed normal white blood cell level (9.6 × 10^9^ /L) and elevated levels of lactate dehydrogenase (266 U/L), procalcitonin (0.46 ng/ml), C-reactive protein (100.23 mg/L), alanine aminotransferase (60 U/L), gamma-glutamyl transferase (238 U/L), and alkaline phosphatase (168 U/L). A CT scan showed multilobar interstitial infiltrates and a small right pleural effusion. Rapid immunochromatographic diagnostic tests for *O. tsutsugamushi* antibodies (Colloidal Gold, WANTAI Inc., China) showed positive IgG and weakly positive IgM. Thus, the patient was diagnosed with scrub typhus complicated by pulmonary involvement and hepatic dysfunction. He was treated with oral doxycycline 100 mg twice daily for 14 days. His symptoms subsided during his five-day hospitalization.

Patient’s DNA samples were isolated from both venous blood and eschar swabs collected on the day of his admission. Nested polymerase chain reaction (PCR) was then performed to amplify the hypervariable regions of the *tsa56* gene using primers described previously [8]. Primers 34 and 55 were used in the first round of PCR with 2 μl DNA template and primers 10 and 11 were used in the second PCR amplification to generate a 408–453 base-pair fragment with 2 μl of the product from the first round as the template. A high-fidelity DNA polymerase (KOD One PCR Master Mix, TOYOBO) was used in all PCR steps in this report. The thermal cycling conditions for both PCRs were 98 °C for 5 min, followed by 30 cycles of 98 °C for 10 s, 58 °C for 5 s, 68 °C for 5 s, and a final elongation step at 68 °C for 1 min. The nested PCR products were sequenced via Sanger sequencing. Its results revealed that the target sequences of blood and eschar samples were identical (GenBank accession no. MZ856313). Sequencing results were also found to be related to the reference *O. tsutsugamushi* Karp genotype (GenBank accession no. LS398548.1) with 99.33% identity at the nucleotide level, and were completely identical to a Karp genotype strain reported in Taiwan (GenBank accession no. MW464199.1). To avoid the sequence variability of the *tsa56* gene affecting primer annealing in some strains, such as some strains of Kato, Shimokoshi and TA686, another pair of primers (5’-AATGTCTGC GTTGTCGTTGCC-3') and (5’-ATAGCGCACACCTACACTTGC-3') were designed to detect nearly full-length *tsa56* coding DNA sequence (CDS) region, based on total 325 sequences of *tsa56* gene collected from the National Center for Biotechnology Information [9, 10] The PCR products were cloned into pLB vectors and the colonies of each sample were randomly selected for Sanger sequencing. The sequencing results have confirmed that there is indeed only one *tsa56* genotype (GenBank accession No. ON568725) via the results of 35 colonies from the blood and 36 colonies from the eschar samples.

Similar to the *tsa56* gene, seven canonical highly conserved genes, as previously selected and reported in the PubMLST database (https://pubmlst.org/), were also subjected to nested PCR amplification, followed by Sanger sequencing [3, 10]. Different from the single *tsa56* genotype, the sequencing results from both the blood and eschar samples exhibited several double peaks at multiple sites in five of the seven conserved genes (*gpsA*, *mdh*, *nrdB*, *ppdk*, and *sucD)* (Fig. 1B–D), which suggests the patient could be co-infected by at least two different *O. tsutsugamushi* strains. To confirm this discovery, the nested PCR products of *gpsA, mdh, nrdB and sucD* were also cloned into the pLB vectors. Thirty to forty colonies of each gene were randomly selected for Sanger sequencing. The sequencing results have showed that it is indeed a co-infection. Both the *gpsA* and *mdh* gene fragments had two different genotypes in the original nested PCR samples (Fig. 1C, D). And both *nrdB* and *sucD* gene fragments had three genotypes, including a major genotype, a minor genotype and another minor genotype mutated from the major genotype (Additional file 1: Fig. S1). There is no significant difference observed in the ratio of the mixed genotypes between the blood and eschar samples, suggesting the evaluations are consistent and the discoveries are very reliable.

Furthermore, the patient’s PCR sequencing results of the conserved genes were compared with all current publicly available typing sequences of 225 *O. tsutsugamushi* isolates from South Korea, Laos, Thailand, Japan, Myanmar, and New Guinea in the PubMLST database [10]. Our results have showed that there were three new single nucleotide polymorphism (SNP) sites discovered that were not reported in any previous sequence types, including two in the *mdh* gene and one in *nrdB* gene. Moreover, we discovered eight new sequence types of alleles that were new combinations of previously reported SNP sites, including one for the *gpsA* gene*,* one for the *nuoF* gene, two for the *mdh* gene, two for the *nrdB* gene and two for the *sucD* gene.

## Discussion and conclusions

The mixed-genotype infection was possibly caused by bites of several chiggers infected with different genotypes of *O. tsutsugamushi* or bites of a single chigger infected with multiple genotypes. Because only one eschar was found in the patient and the ratio of mixed genotypes of conserved genes was consistent between the eschar and blood samples, the mixed-genotype infection in this patient was possibly caused by bites of a single chigger infected with multiple *O. tsutsugamushi* genotypes. Interestingly, mixed genotypes of *O. tsutsugamushi* in conserved genes have been reported in approximately 25% of patient blood samples in Thailand [3]. In contrast, although a mixed-genotype of the *tsa56* gene was found in 17.9% of 28 chiggers [11], few mixed infections have been reported in patients [5–7]. A possible explanation of this observable difference could be response to the reservoir host immune selection pressure and a high rate of intraspecific recombination in the intracellular bacteria *O. tsutsugamushi*[12]. This assumption surely needs to be investigated on a large scale specifically focusing on the rates of mixed infections from both *tsa56* and the conserved genes.

Meanwhile, the sequencing results from blood and eschar samples repeatedly showed 32 variants happened in 2700 base-pair long and concatenated sequence region of conserved genes, whereas no variant was discovered in the *tsa56* gene. The discovery on the single *tsa56* gene here is very interesting, considering the fact that the tsa56 gene has multiple hypervariable regions where variants are supposed to be discovered more frequently than the highly conserved genes. The exact mechanism behind this mixed-infection case is unknown. One possibility could be ancestor of the minor strain might carry with a different *tsa56* genotype and became extinct at some point in one population under the reservoir host immune selection pressure. Some genotypes of conserved genes originally in the extincted strain were probably preserved due to high frequency recombination events occurring in *O. tsutsugamushi*.

This mixed-infected patient was successfully recovered by the doxycycline treatment. Yet, the correlation between the single-tsa56-genotype mixed infections and disease severity remains unclear and would be evaluated at a large scale with more cases. Moreover, the ratio of the mixed genotypes of conserved genes was consistent between the eschar and blood samples, suggesting the discovery is very reliable and, meanwhile, eschar samples also can be utilized to detect minor genotypes in *O. tsutsugamushi* infections.

## Supplementary Information

**Additional file 1: Figure S1.** Mixed genotypes of O. tsutsugamushi identified from both eschar and blood samples of the patient. **A** and **B** The results of direct sequencing of PCR amplicon and colony verification of nrdB and sucD genes. These sites are all verified by sequencing of 30-40 clones for each gene and each sample. **C** The results of direct sequencing of PCR amplicon of ppdk gene. The logo presents the nested PCR fragments of nrdB, sucD and ppdk genes collected in the pubMLST database. Mixed sites are indicated by filled arrows. These mixed sites are either synonymous (light blue letters) or missense (red letters).

## Data Availability

All sequences analyzed during this study are available from the NCBI database (accession numbers MZ856313, ON568725-ON568737).

## Abbreviations

*O. tsutsugamushi*: *Orientia tsutsugamushi*
*tsa56*: 56-KDa type-specific antigen
SNP: Single nucleotide polymorphism
MLST: Multilocus sequence typing
PCR: Polymerase chain reaction
CDS: Coding DNA sequence
