# Supplementary material for: Mixed genotypes of Orientia tsutsugamushi in conserved genes and a single immune-dominant tsa56 genotype discovered from a patient with scrub typhus in Hainan Island, China: a case report
Source: BMC Infect Dis. 2022 Aug 19;22:698. doi: 10.1186/s12879-022-07682-y (PMC9389790; doi:10.1186/s12879-022-07682-y)

**Additional file 1:** Mixed genotypes of *O. tsutsugamushi* identified from both eschar and blood samples of the patient. A) and B) The results of direct sequencing of PCR amplicon and colony verification of *nrdB* and *sucD* genes. These sites are all verified by sequencing of 30-40 clones for each gene and each sample. C) The results of direct sequencing of PCR amplicon of *ppdk* gene. The logo presents the nested PCR fragments of *nrdB,* *sucD* and *ppdk* genes collected in the pubMLST database. Mixed sites are indicated by filled arrows. These mixed sites are either synonymous (light blue letters) or missense (red letters).


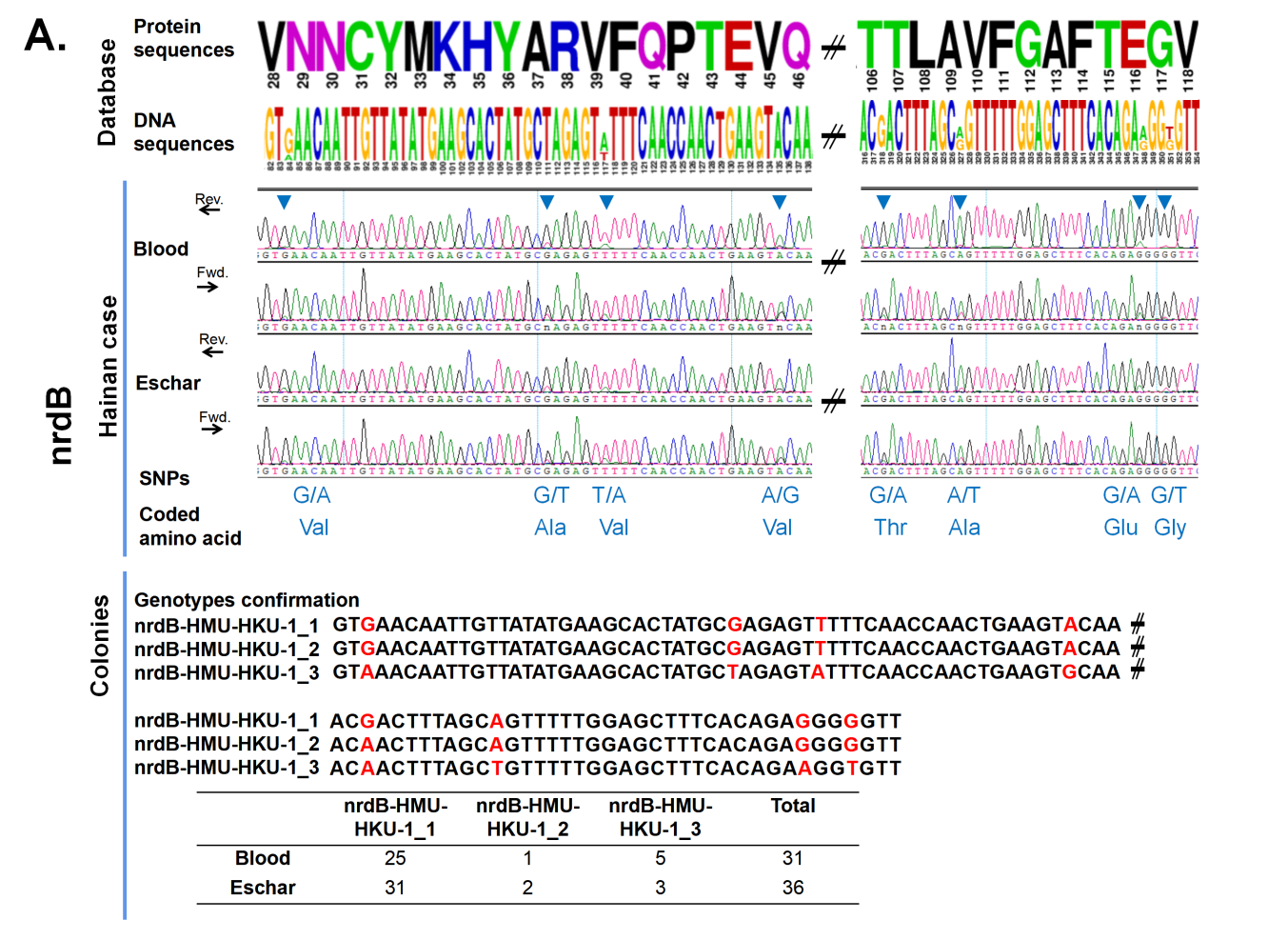


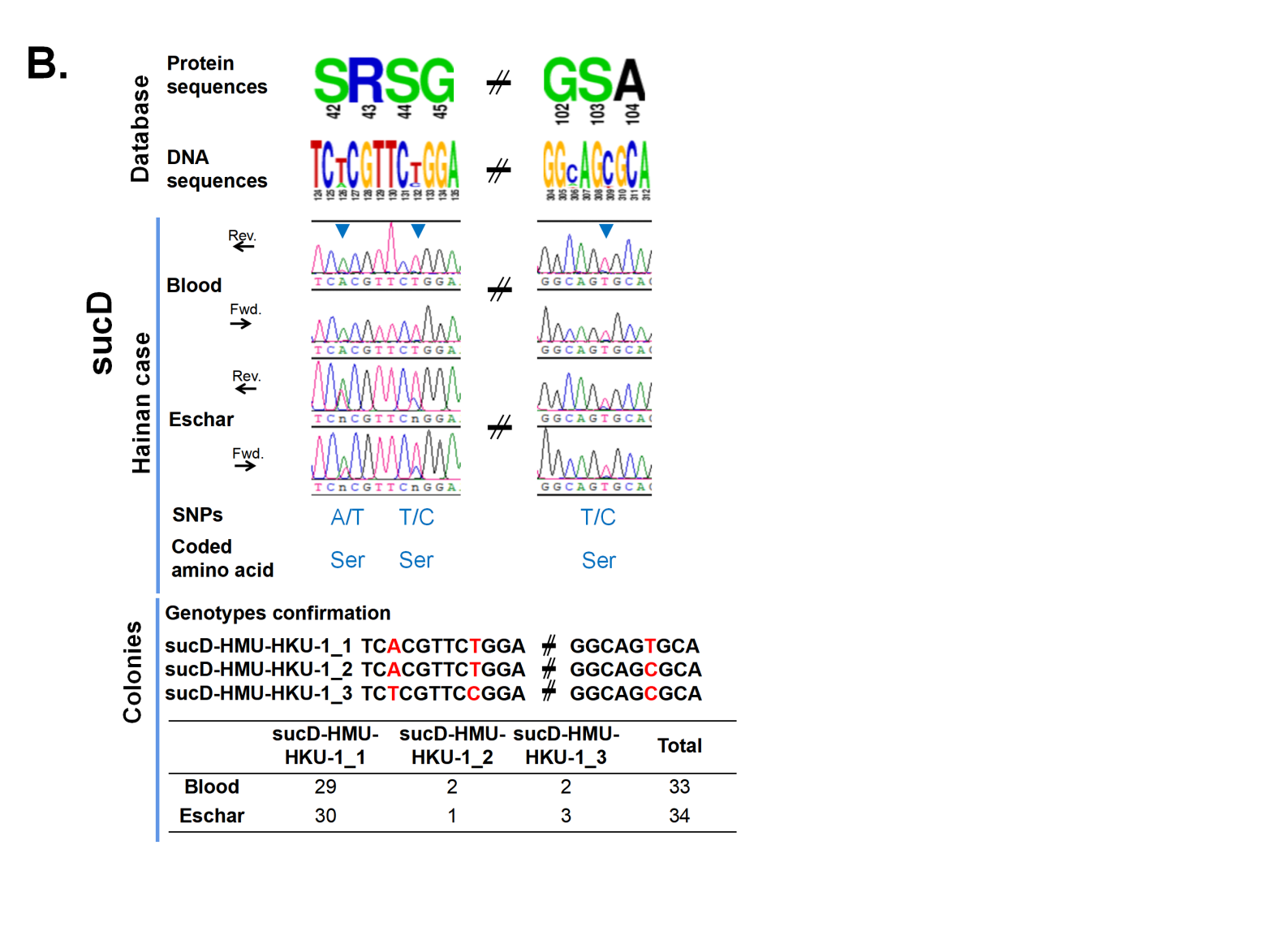


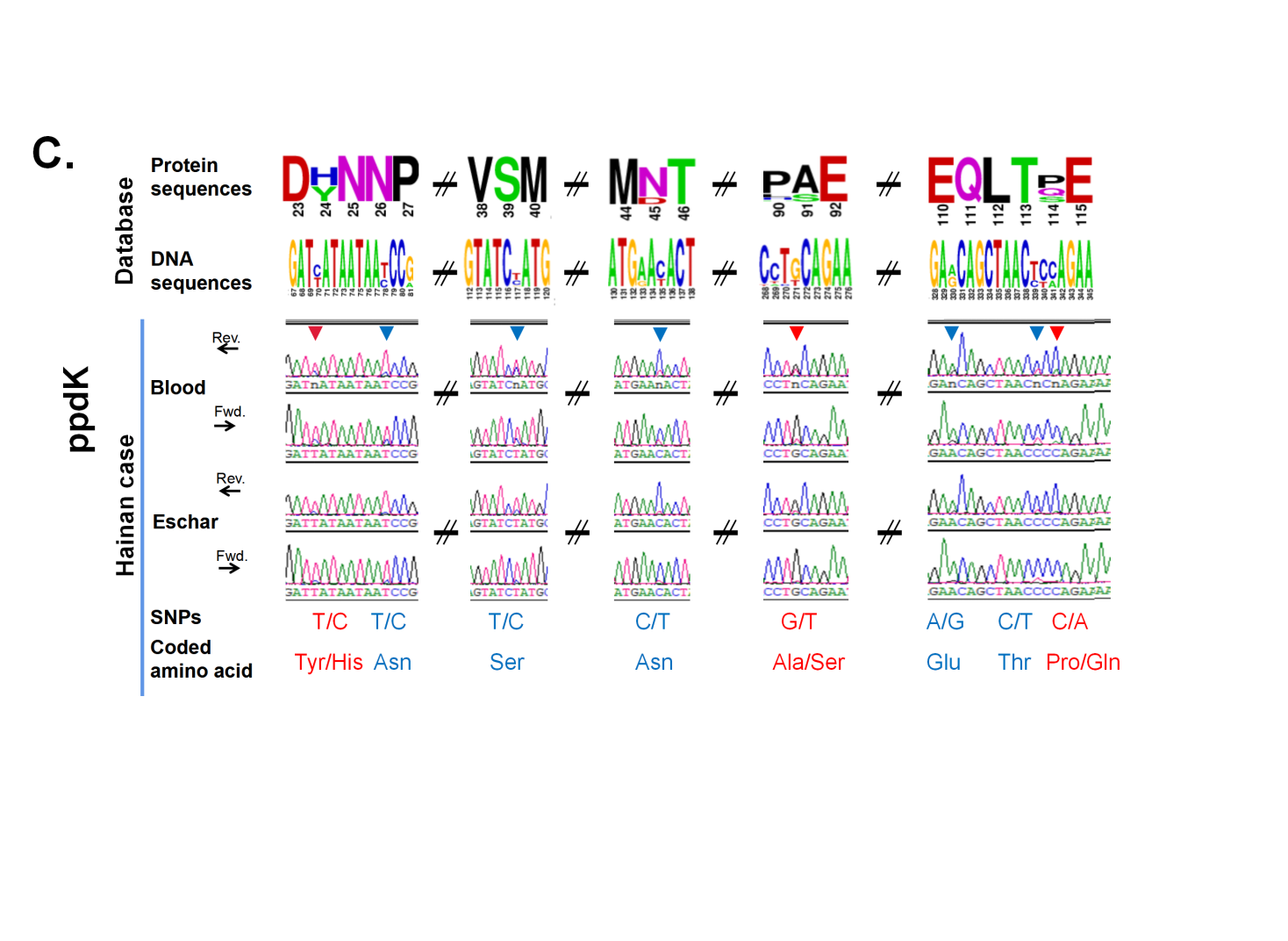

Supplement: Supplementary file 1 — Additional file 1: Figure S1. Mixed genotypes of O. tsutsugamushi identified from both eschar and blood samples of the patient. A and B The results of direct sequencing of PCR amplicon and colony verification of nrdB and sucD genes. These sites are all verified by sequencing of 30-40 clones for each gene and each sample. C The results of direct sequencing of PCR amplicon of ppdk gene. The logo presents the nested PCR fragments of nrdB, sucD and ppdk genes collected in the pubMLST database. Mixed sites are indicated by filled arrows. These mixed sites are either synonymous (light blue letters) or missense (red letters). [file 12879_2022_7682_MOESM1_ESM.docx]
